# Traces of EEG-fMRI coupling reveals neurovascular dynamics on sleep inertia

**DOI:** 10.1038/s41598-024-51694-4

**Published:** 2024-01-17

**Authors:** Zhitong John Wang, Hsin-Chien Lee, Chun-Hsiang Chuang, Fan-Chi Hsiao, Shwu-Hua Lee, Ai-Ling Hsu, Changwei W. Wu

**Affiliations:** 1 Graduate Institute of Mind, Brain and Consciousness, Taipei Medical University, 5 Floor, 301, Yuantong Rd., Zhonghe Dist, New Taipei, 235040 Taiwan; 2 Department of Psychiatry, School of Medicine, College of Medicine, Taipei Medical University, Taipei, Taiwan; 3 Research Center of Sleep Medicine, Taipei Medical University Hospital, Taipei, Taiwan; 4 Research Center for Education and Mind Sciences, College of Education, National Tsing Hua University, Hsinchu, Taiwan; 5 Department of Counseling, Clinical and Industrial/Organizational Psychology, Ming Chuan University, Taoyuan, Taiwan; 6 Department of Psychiatry, Chang Gung Memorial Hospital at Linkou, 259, Wenhua 1St Rd., Guishan Dist., Taoyuan, 33302 Taiwan; 7 School of Medicine, College of Medicine, Chang Gung University, Taoyuan, Taiwan; 8 Bachelor Program in Artificial Intelligence, Chang Gung University, Taoyuan, Taiwan

**Keywords:** Neuroscience, Physiology, Medical research

## Abstract

Upon emergence from sleep, individuals experience temporary hypo-vigilance and grogginess known as sleep inertia. During the transient period of vigilance recovery from prior nocturnal sleep, the neurovascular coupling (NVC) may not be static and constant as assumed by previous neuroimaging studies. Stemming from this viewpoint of sleep inertia, this study aims to probe the NVC changes as awakening time prolongs using simultaneous EEG-fMRI. The time-lagged coupling between EEG features of vigilance and BOLD-fMRI signals, in selected regions of interest, was calculated with one pre-sleep and three consecutive post-awakening resting-state measures. We found marginal changes in EEG theta/beta ratio and spectral slope across post-awakening sessions, demonstrating alterations of vigilance during sleep inertia. Time-varying EEG-fMRI coupling as awakening prolonged was evidenced by the changing time lags of the peak correlation between EEG alpha-vigilance and fMRI-thalamus, as well as EEG spectral slope and fMRI-anterior cingulate cortex. This study provides the first evidence of potential dynamicity of NVC occurred in sleep inertia and opens new avenues for non-invasive neuroimaging investigations into the neurophysiological mechanisms underlying brain state transitions.

## Introduction

Sleep and wake are distinct conscious states that differ in electrophysiology and phenomenal experiences, and the transition from sleep to wake is often accompanied by a state of grogginess and disorientation called sleep inertia, lasting for several minutes to hours after awakening^1–3^. Sleep inertia is therefore regarded as a risk factor for professionals who need to immediately begin work after waking up^4,5^. Despite its prevalence in everyday life, sleep inertia has received less attention in research compared to sleep and wakefulness, and its exact neurophysiological mechanism behind the transient vigilance recovery is unknown^3^. Most neuroimaging studies on sleep inertia have been limited to EEG and fMRI, comparing pre-sleep and post-sleep states. Upon awakening, EEG activity shows elevated delta power and reduced beta power compared to pre-sleep^6,7^. Recent research identified EEG-frequency spectral slope (also known as “1/*f*” component) as an alternative EEG marker of human arousal in REM that is diminished in comparison to wakefulness, suggesting that unique neural activities underlying REM could be captured via electrophysiology^8,9^. The theta/beta ratio is another promising candidate that approximates attentional control cognitive processing capacity, which might provide insights into the transient fluctuations of cognitive states^10–12^. On the other hand, fMRI-based functional connectivity analysis has identified reduced inter-hemispheric connectivity within the sensorimotor (SM) network and increased connectivity between the thalamus and neocortex after awakening from nocturnal sleep^13^. Additionally, a decrease in anticorrelation between the default-mode network (DMN) and task-positive networks (including the dorsal-attention, salience, and somatosensory networks) was observed after awakening from an afternoon nap^14^. In an EEG-fMRI fusion study, reduced correlation between the frontal-parietal network and an EEG-based vigilance index, alpha-vigilance, was found during sleep inertia, where the alpha-vigilance was defined as the alpha power (0.5–12 Hz) divided by theta and delta power^15^. Overall, these findings provide evidence that sleep inertia is a unique brain state characterized by altered functional network reorganizations and electrophysiology vigilance indices.

Recent neurovascular coupling (NVC) investigation in humans has been made possible with the development of various neuroimaging techniques on the basis of local hemodynamics during neural activity^16^. Above-mentioned EEG-fMRI studies on sleep inertia have assumed constant NVC throughout the course of awakening, while there is growing evidence that NVC is dependent on brain states. In particular, past studies on EEG spectral amplitudes during sleep have found that delta power increases as sleep stage deepens from N1 to N3^17^, but fMRI fluctuation amplitude reached its maximum at N2 instead^18^. Furthermore, bilateral fMRI connectivity exhibited spatial mismatch with interhemispheric EEG delta connectivity, suggesting a dynamic NVC change during sleep in a macroscale scope^18^. This phenomenon could be due to cerebral spinal fluid (CSF) oscillation during non-rapid-eye-movement (NREM) sleep, which is coupled to delta oscillations and anticorrelated to BOLD oscillation only during sleep but not during wake^19^. Gu et al. also showed that the correlation between occipital alpha power and BOLD activity in the thalamus, dorsal anterior cingulate cortex (dACC) and SM cortex are different across various time-lags depending on eyes-open or eyes-closed condition, suggesting altered NVC that is dependent on arousal fluctuations^20^. Computational modeling also demonstrated that neuronal synaptic activity may be a greater contributor to BOLD signal than population spiking rate when overall neuronal activity is low^21^, which highlighted that NVC could be dynamic and dependent on specific neural circuitry mechanisms.

**Figure 1:**
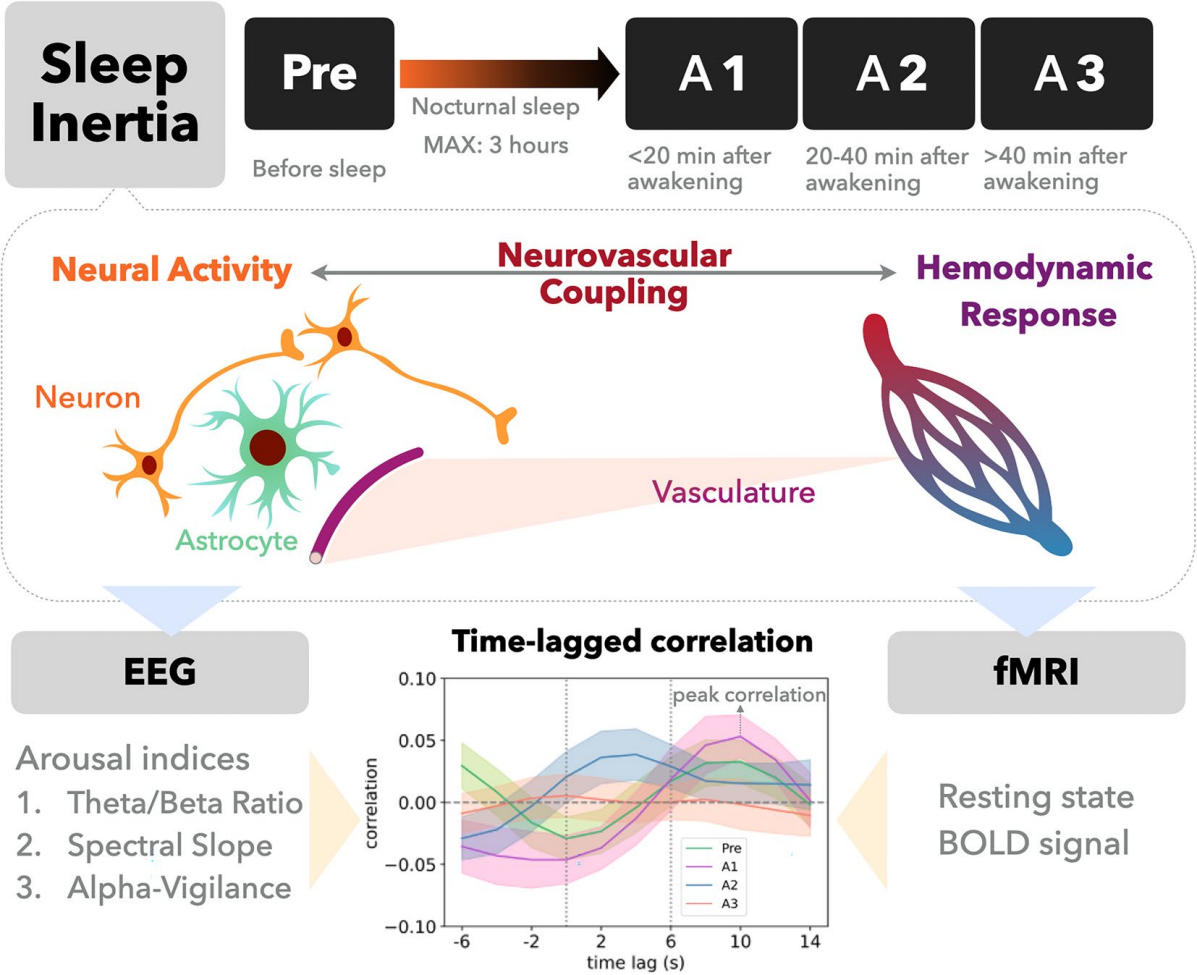
Illustration of experimental design and analytical indices. During sleep inertia, the neurovascular coupling may change along with this recovery period of consciousness. We probed the transitory changes of EEG-fMRI coupling, surrogates of neurovascular coupling, depending on the extended awakening time (A1 to A3).

Generally, the majority of NVC investigations is based on microscopic biophysiological evidence, but in the human brain, NVC is a neurophysiological concept rather than a quantifiable index in the macroscopic neuroimaging research^16^. However, to highlight the NVC dynamics in sleep inertia, a quantified index for NVC is mandatory, otherwise presenting temporal consistency from each component of neurovascular units provides relatively weak evidence to clarify the underlying NVC changes in normal participants^22^. Therefore, building off recent discoveries from time-lagged neurovascular activity analysis^20,23^, we applied it to examine NVC dynamics during sleep inertia from multiple EEG markers of cognitive and arousal states and their associations with BOLD signals in related brain regions. Specifically, we aimed to probe the coupling dynamics between BOLD signals and EEG features of brain states across the initiation and dissipation of sleep inertia through simultaneous EEG-fMRI recordings. Three ROIs were prescribed of particular interest: **thalamus** for arousal modulation through the thalamo-cortical pathway^24–26^, **ACC** for regulating salience and vigilance^27,28^, and **SM cortex** for its broad coverage of the cortical layers and potential implications in sleep inertia^13^. To elucidate the precise mechanism behind the initiation, maintenance, and dissipation of sleep inertia, we computed (1) EEG spectral slope between 1 and 45 Hz, (2) alpha-vigilance, and (3) theta-beta ratio, assessed EEG state changes across three imaging sessions post-awakening, and finally examined the coupling dynamics between EEG-state variables and BOLD signal time course. Figure 1 illustrates the concept of neurovascular coupling, as well as the experimental design and the analyses in this work.

## Materials and methods

### Participants

Twenty-one healthy subjects (age: 26.6 ± 4.18, 10 females) participated in this study. All subjects reported no neurological and psychiatric disorders, and were given the Pittsburgh Sleep Quality Index (PSQI) to assess for any sleep disturbances over a one-month period before experimentation. Subjects were then asked to maintain a consistent sleep–wake schedule, monitored through wrist actigraphy (SOMNOwatchTM plus, SOMNOmedics GmbH, Randersacker, Germany), for more than three days prior to experiment. Through the actigraphy records, we could confirm that all participants maintained a consistent sleep–wake cycle, which enabled us to determine the habitual sleep time for each participant. All methods were performed in accordance with the relevant guidelines and regulations by the National Yang-Ming University University Institutional Review Board (YMeIRB, Approval No. 201512ES054). All participants provided written consent to the study procedures.

### Experimental procedure

Participants who passed the initial screening process underwent nocturnal sleep inside the MR scanner while wearing a MR-compatible EEG cap (detailed below). The participants were instructed to lie supine in the MRI scanner after EEG-cap preparations. No caffeine and alcohol usage was permitted on the day of experiment. The procedure was summarized in Fig. 1. Before the subject's normal sleep time, a 6-min T_1_-weighted anatomical scan was acquired, followed by a 5-min eye-open resting state fMRI scan (rs-fMRI; pre-sleep) and then subjects underwent sleep (maximum of 3 h of sleep). Participants were asked to fall asleep after the scan started, and the termination criteria for the sleep session were (1) a scan time reached a predefined 180-min limitation and we asked the participant to wake up; or (2) a participant who felt unable to fall asleep for any extended period was instructed to terminate the session through pressing the alarm ball. Upon awakening, participants were instructed to maintain body position still and continuously underwent three 5-min rs-fMRIs with 20 min interscan intervals (A1, A2, A3, respectively). Psychomotor-vigilance task and breath-hold cerebrovascular reactivity data were also collected within the 20-min interscan intervals and reported elsewhere^22^.

### Simultaneous EEG-fMRI acquisition

Subjects underwent anatomical scans during the ‘Pre’ stage, where their T_1_-weighted images were collected using a 3 T Siemens Tim Trio system (Erlangen, Germany) with 3D-MPRAGE sequence (TR/TE/TI = 1900 ms/2.28 ms/900 ms, FA = 9°, 176 slices, 1 × 1 × 1 mm^3^) in National Yang-Ming University. The rs-fMRI in ‘Pre’, ‘A1’, ‘A2’ and ‘A3’ were then acquired using gradient-echo EPI sequence (TR/TE = 2000 ms/30 ms, FA = 77°, 32 slices, slice thickness of 4 mm with no gap, voxel size of 3.44 × 3.44 × 4 mm^3^).

EEG data was acquired concurrently during the MR sequence with a MR-compatible, 32-electrode EEG system (Brain Products, Gilching, Germany). The EEG system included two electrooculography (EOG) channels, one electrocardiogram (ECG) channel, and two electromyography (EMG) channels. For reference and ground channels, FCz and AFz channels were selected, respectively. Electrodes’ impedances were kept below 15 kΩ using abrasive electrode paste (ABRALYT HiCl), with the reference and ground channels kept below 5 kΩ. Raw EEG signals were recorded using the BrainVision recorder (Brain Products) with 5000 Hz sampling rate and 0.1-μV voltage resolution. An analog band-pass filter (0.0159–250 Hz) and a 60-Hz notch filter were then applied to reduce machinery and extraneous noises. EEG signals were then synchronized with MR triggers using Brain Products Trigger Box and the software of E-Prime Extensions for Brain Products.

### Data processing

We used the CONN21.a toolbox^29^ implemented in MATLAB to conduct all preprocessing: realignment and unwarp, segmentation of gray/white matter and cerebral spinal fluid, smooth with a 6 mm full width at half maximum (FWHM) Gaussian kernel, and registered to the Montreal Neurological Institute (MNI) space. Next, nuisance factors (linear trends, CSF signals, white matter signals, and motion) were regressed out.

EEG data was first upsampled to 50,000 Hz for gradient artifact and ballistocardiogram-induced artifacts removal using averaged artifact subtraction in Brain Vision Analyzer 2.1 (Brain Products) and downsampled to 250 Hz. EEG data was then processed in EEGLAB (v13.6.5b) with re-referencing, epoching around each MR trigger (2 s epochs), bad epoch removal using the ‘autorej’ function in EEGLAB (an average of 14.82 ± 3.89 bad epochs out of 150 per session were rejected), and removal of additional physiological noises using independent component analysis through manual inspections^30^. The spectral power for each electrode was computed using the psd_multitaper function in MNE-python (v0.24.1) with default settings (7 DPSS windows with half-bandwidth of 4 Hz)^31^. The FOOOF (v1.0.0) package was used to calculate the spectral slope between 1 and 45 Hz with peak width between 1 and 8 Hz^32^. Alpha-vigilance was derived by taking the ratio of the absolute power of the alpha band (8–12 Hz) with the average of absolute theta (4–8 Hz) and delta band (0.5–4 Hz) power^15^. Theta/beta ratio was calculated by dividing the absolute theta band power by absolute beta band (12–25 Hz) power. Data in noisy epochs marked by EEGLAB’s ‘autorej’ function were excluded, and clean epochs that surrounded noisy epochs were instead used to interpolate noisy epochs’ EEG features via the ‘interpolate.interp1d()’ function in SciPy library in Python (v1.9.1). Epoched EEG feature time series were then averaged across all electrodes for each subject for correlation analyses with BOLD signals. To assess for changes in EEG features magnitude across imaging sessions, the EEG feature time series were instead averaged across all electrodes and epochs to generate a single value per imaging session.

### Automatic sleep scoring

All participants’ sleep stages and durations were automatically scored using YASA (v0.6.3), an automated sleep-staging algorithm trained and validated on ~ 30, 000 h of polysomnography (PSG) to achieve comparable accuracy to human sleep scorers^33^. Following original authors’ recommendations, EOG, EMG and EEG channel ‘C4’ after gradient artifact removal, downsampling to 100 Hz, and filtered between 0.4 and 30 Hz were used by YASA to generate hypnograms with 30 s epochs for all subjects. One sleep technician additionally validated and modified incorrectly scored epochs (< 1% of totally scored epochs).

### Cross-correlation and ROI selection

Three ROIs were investigated for this study: thalamus, ACC, and SM cortex. The thalamus region was defined using the Harvard–Oxford subcortical atlas, ACC was defined using the Harvard–Oxford cortical atlas^34^, and the SM cortex was defined using the Juelich histological atlas^35^ all at maximum probability thresholded at 25%. Voxels in each ROI were averaged and extracted as a time series using the Nilearn-Python (v0.9.2) package. The ROI-based fMRI time course was extracted by taking the averaged BOLD signal in all associated voxels per ROI (Figure S1A). EEG features were computed in 2-s epochs to match the time resolution of BOLD time series for cross-correlation (Figure S1B). The timing and magnitude of maximum correlation and anticorrelation across a selected range of time-lags were compared (Figure S1C,D).

### Statistical analysis

Nonparametric repeated-measure Friedman tests were performed across the four consecutive sessions (Pre, A1–A3), followed by post hoc tests of Wilcoxon sign-rank test with false discovery rate (FDR) correction, using Python (SciPy version 1.9.1) to determine significant differences. *P*-value < 0.05 was considered to be significant.

### Ethical statement

The study protocol was approved by the Research Ethics Committee of National Taiwan University (No. 201512ES054). All participants provided their written informed consent to participate in this study.

## Results

### EEG features across conditions

**Figure 2:**
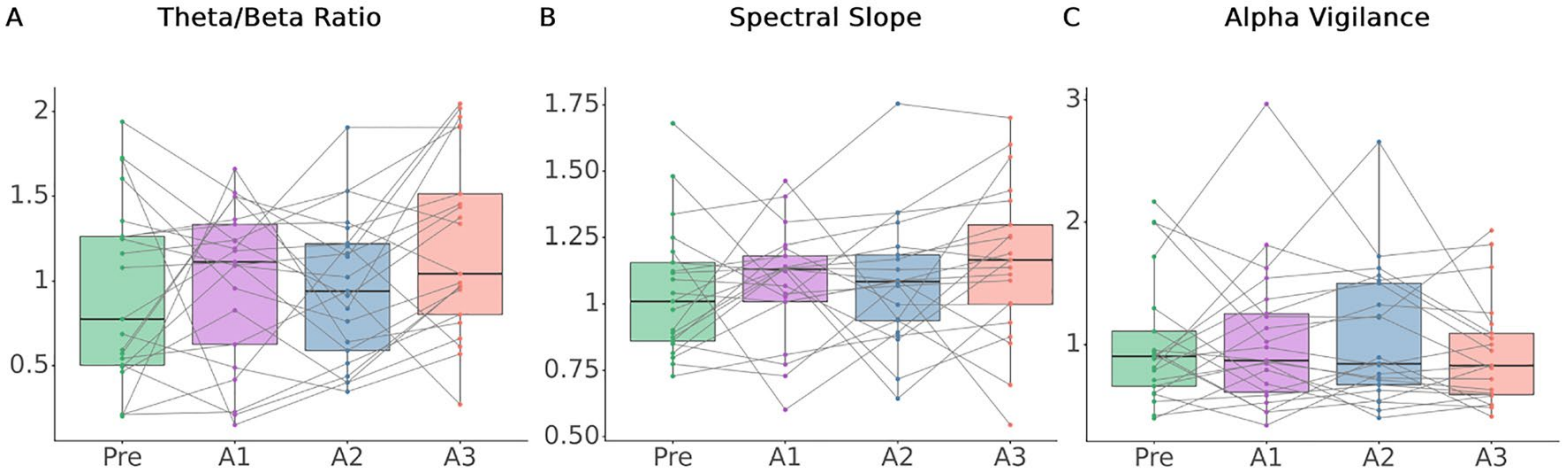
EEG arousal features across four imaging sessions. (**A**) Averaged theta/beta ratio per imaging session per each subject was used to compare across four imaging sessions (Friedman test *p* = 0.09). Theta/beta ratio in A3 is higher than A2 but without statistical significance (Wilcoxon sign-rank test *p* = 0.06, FDR-corrected). (**B**) Spectral slope was found to fluctuate significantly across imaging sessions (Friedman test *p* = 0.025), but no pairs of imaging sessions were found to be significantly different. (**C**) No significance was found for alpha-vigilance across imaging sessions (Friedman test *p* > 0.1).

From the sleep scoring (Table S1), we confirmed that all participants fell asleep in the sleeping session, and the sleep stages before awakening were listed below: 6 from N1 sleep (27%); 9 from N2 sleep (41%); 3 from N3 (14%) and 3 from REM sleep (14%). Our first aim was to probe whether proximate measurement of cognitive and arousal states through EEG features can reflect the initiation and dissipation of sleep inertia. We assessed whether theta/beta ratio, EEG spectral slope, and alpha-vigilance varied across imaging sessions during sleep inertia. We found no significance across four sessions in theta/beta ratio (Friedman test, *p* = 0.09; Fig. 2A). There was a significant effect by imaging session for spectral slope (Friedman test, *p* = 0.025), however, in lack of significance in post hoc tests (*p* > 0.1, FDR-corrected; Fig. 2B). Alpha-vigilance did not significantly change across imaging sessions (Friedman test, *p* > 0.1; Fig. 2C).

### ROI selection and correlation time course

Considering sleep inertia’s relative quick dissipation and potential fluctuations of hemodynamic response during brain state transitions, it was therefore necessary to conduct an analysis that could address the temporal relationship between electrophysiological and BOLD-fMRI signals simultaneously. Specifically, we wanted to assess whether the coupling between EEG features and ROI-based fMRI activity fluctuated across imaging sessions. The correlation values were found to fluctuate across extended time lags in periodic fashion (Figure S1C), perhaps due to intrinsic autocorrelation within each signal ^36–38^. We followed up with averaged periodogram plots of the correlation values depicting dominant frequencies residing mostly between the range of 0.01–0.1 Hz with frequency averaging around 0.05 Hz (Figure S2). The 0.05 Hz frequency pointed to the optimal time-lag range to examine when EEG and fMRI signals exhibit closest coupling between each other within one period, therefore minimizing the influence of multiple correlation peaks (Figure S1C). Furthermore, this periodicity pattern remained consistent across EEG metrics, allowing us to choose one frequency range for all EEG features.

### Peak-correlation time lag comparison across conditions

**Figure 3:**
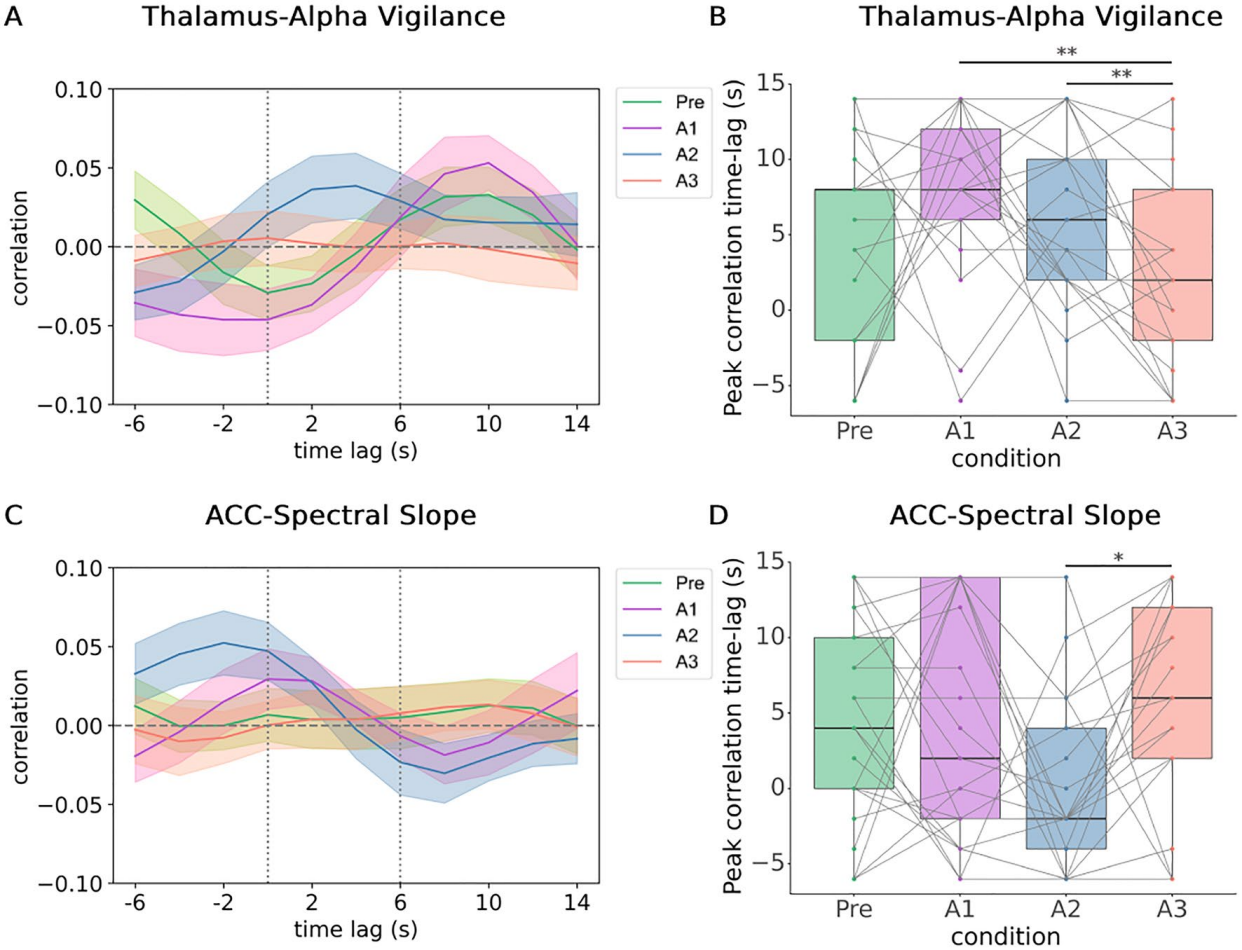
Time lag of peak positive correlation among sessions across sleep inertia. (**A**) Time lags where maximum positive correlation between thalamus and alpha-vigilance occurs across a [− 6, 14] s range were plotted for each subject. (**B**) There is a significant effect by imaging session (*p* = 0.02), where A1 and A2 were found with peak-correlation timings that are significantly later than A3 (**p = 0.01, FDR-corrected). (**C**) Time lags where maximum positive correlation between thalamus and EEG spectral slope occurs across a [− 6, 14] s range. (**D**) There is a significant effect by imaging session (*p* < 0.01), where A2 is found to be significantly earlier than A3 (*p = 0.03, FDR-corrected).

Considering the average frequency of the cross-correlation functions being 0.05 Hz, we chose the − 6–14 s time range to maximally capture meaningful fluctuations in EEG features and BOLD signals coupling (Figure S1D, Figure S3). Then, we assessed the time lag at which peak correlation (maximum correlation coefficient) and peak anti-correlation (minimum correlation coefficient) occurred within this time range between each EEG feature and BOLD ROI (Figure S4). We found a significant effect by imaging session in peak positive correlation timing between alpha-vigilance and thalamus activity (Friedman test, *p* = 0.025), and post-hoc test identified a significant difference between A1 and A3, as well as A2 and A3 (*p* < 0.01, FDR-corrected; Fig. 3A,B). Imaging session also had a significant effect in peak positive correlation timing between spectral slope and ACC activity (Friedman test, *p* < 0.01). Post-hoc test found that A2’s peak-correlation timing was significantly earlier than A3 (*p* = 0.03, FDR-corrected), and there was a shift to an earlier time-lag from ‘Pre’ to ‘A2’, and restored to ~ 6 s time-lag in A3 (Fig. 3C,D). There was no significant difference in peak-anticorrelation timing, as well as magnitude of maximum correlation and anticorrelation values across imaging sessions (Figure S3).

## Discussion

NVC is a general presumption in neurophysiology, but accumulating literature indicates that NVC may not be constant, and is subject to changes depending on a person’s age^39^, health^40–42^, cerebral blood flow (CBF) and ongoing neural activity ^43^. Our study is the first investigation of sleep inertia that evaluated multiple EEG metrics of brain states and EEG-BOLD coupling dynamics to probe the NVC alterations across three imaging sessions (A1-A3) after awakening. If the NVC remains unchanged during sleep inertia, we assume that both the amplitude and time lags between EEG metrics and BOLD-fMRI responses remain time-invariant from A1 to A3. In the results, the time-varying amplitudes and lags of the EEG-fMRI coupling provided preliminary evidence of an altered NVC during sleep inertia. From literature, both electrophysiology and hemodynamic responses experience dramatic alterations during sleep inertia. In EEG, reduced delta and enhanced beta power has been reported^6,15^, and these metrics change along the waking time^14^; similarly, the cerebral blood flow (CBF) is slower after sleep for up to 30 min than pre-sleep^44,45^. Given the mismatches of recovery speed between the two neurophysiological mechanisms, electrophysiology and hemodynamics, during sleep inertia, it is likely to drive NVC away from its normal relationship, reflected on the altered EEG-fMRI relationship in arousal-related brain regions. This dynamic process resembles the EEG-fMRI relationship during the NREM sleep^18^, which is another transitory condition with significant neurophysiological variations. From our study, we observed relatively long time-lags (6 ~ 8 s) of peak-correlation between alpha-vigilance and BOLD-thalamus during ‘A1’ and ‘A2’. As wakefulness prolongs, cortical inhibitory interneurons resume the more significant role in regulating arousal through innervating cortical microcircuits and generating high frequency oscillations such as gamma activity^25,46^. NVC is therefore again altered, and peak-correlation time lag between alpha-vigilance and BOLD-thalamus returns to ~ 2.5 s as reported by past literature^47^. Chen et al. reported no significant correlation between alpha-vigilance and BOLD-thalamus between pre-sleep and post-awakening ^15^, which might be due to the dynamic feature of NVC in sleep inertia, rather than a static coupling that can be probed by single measure after awakening.

The altered EEG-fMRI relationship is also dependent on brain regions. In the current study, we probed three brain regions relevant to arousal fluctuations according to Gu et al., and only found the time-lags were time varying in EEG alpha vigilance—BOLD thalamus and EEG spectral slope—BOLD ACC^20^; however, the cortical sensorimotor areas did not present the alterations of EEG-fMRI coupling (Figure S4 C, F, I). Based on previous investigations on hemodynamics, Balkin et al. reported that the thalamus and brainstem were the first regions to restore normal CBF upon awakening ^48^, and the fast CBF into thalamus immediately upon awakening is important for the thalamus conducting arousal signaling to the cortex through the calbindin1-positive matrix cells in its ventromedial nucleus^49,50^. Recently, Setzer et al. used the fast fMRI (TR = 400 ms) and observed the fast thalamus and ACC activities before the behavioral arousal, but the cortical regions showed deactivation after post-sleep arousal, which partly supports the functional distinctions between the thalamus/ACC versus other cortical regions^51^. Furthermore, Setzer et al. also addressed that the thalamic functional change from burst to tonic firing during the transient arousal may come with uncommon hemodynamic redistributions to more active areas^51^, which was in agreement with our speculation of the mechanisms underlying the observed NVC alterations.

### Quantification of NVC dynamics

The initial concept of BOLD principle and NVC was built from the brain states under task engagements with clear identification of neural activity and follow-up hemodynamic response function (HRF). Relatively, examining NVC through the resting-state paradigm is difficult, as there are no neural events time locked to behavior, making it challenging to deconvolve the HRF for direct comparison across sessions. Nevertheless, there is growing evidence that NVC may fluctuate depending on distinctive brain states, and HRF is not stable^18,52–56^. Past research has found that the correlation between neural activity and cerebral blood volume varies across the sleep–wake cycle, seeing stronger coupling during NREM in comparison to REM sleep and wake^23^. We adopted a similar approach that used cross-correlation to examine the coupling between BOLD and EEG metrics and reported on NVC variations along the sleep inertia period. A recent EEG-fMRI study reported that brain signals in the salience network declines approximately 6 s (12 s with typical hemodynamic delay) prior to the occipital alpha decreases^20^. This suggested that BOLD signal fluctuations may lead EEG signals for up to 6 s during transient arousal fluctuations, which led us to choose the time window between − 6 and 14 s in an attempt to maximally capture relevant fluctuations between electrophysiological and hemodynamic signals related to arousal. Furthermore, there is evidence showing that EEG-BOLD correlations during resting-state might mostly be due to transient arousal modulations, which in turn causes sequential alterations of EEG spectra across a ~ 20 s period^57^. We therefore chose the time window between [− 6, 14] to maximally capture related neurophysiological information and assess when peak correlation occurs. Consequently, we found that during this transition period from sleep to wakefulness, NVC fluctuations were observed with respect to the alpha-vigilance and BOLD-thalamus as well as EEG-spectral slope and BOLD-ACC. Importantly, we chose not to convolve the EEG metric time series by HRF, as there is insufficient evidence suggesting a direct consequence between EEG indices (vigilance index and spectral slope) and HRF during sleep inertia prior to our study. Furthermore, spontaneous hemodynamic signal during resting-state might not only be caused by neural activity but also micromovements and non-neural processes as well^20,58^, creating additional uncertainty for the consistent NVC assumption in sleep inertia. The EEG metrics evaluated in this study are considered as brain state variables explained by electrophysiological signals, and their temporal correlation with BOLD signals may serve as an alternative to assess transient NVC changes without being limited to uncertain and dynamic HRF during sleep inertia.

### EEG metrics and BOLD coupling in sleep inertia

Previous research on EEG-BOLD coupling primarily focused on elucidating the relationship between EEG spectral power and fMRI functional connectivity or brain regional activity. For instance, alpha power, primarily from the occipital region, has been shown to anticorrelate with BOLD signal in the occipital, frontal, temporal, and parietal cortex, while having a positive correlation with thalamic BOLD signal, all under the assumption of the ~ 6 s canonical hemodynamic response delay^20,59,60^. Similarly, alpha-vigilance has been reported to show a similar pattern^61^, suggesting that cortical electrophysiological metrics are closely related to hemodynamic activity in related brain regions under wakeful resting states. While the precise modulation mechanism of arousal during sleep inertia is unclear, the transition from low arousal during sleep to high arousal during wake might involve different modulation mechanisms. The thalamo-cortical pathway is state dependent where inhibition/excitation shifts between sleep and wakefulness^25^. Upon awakening, the thalamus receives input from wakefulness-promoting regions such as locus coeruleus^62^, tuberomammillary nucleus^63^, and dorsal and median raphe nuclei^64,65^. Thalamo-cortical synchronization is also enhanced upon awakening^13^; however, sleep-promoting mechanisms such as cortical interneurons that produce nitric oxide synthase might still remain active to inhibit arousal-promoting regions^66–68^, which in turn results in the groggy and sleepy feeling during sleep inertia that aims to protect sleep from undesired awakenings^3,69^. This may explain our findings on peak-correlation timing between alpha-vigilance and thalamus immediately upon wake (‘A1’), where the positive correlation between the thalamus and alpha-vigilance is significantly delayed to ~ 8 s, much later than the ~ 2.5 s lag described by previous literature^47,61^. Since it is possible to have simultaneous activation in both sleep-promoting (cortical) and wake-promoting (subcortical) mechanisms during this transitory brain state, the peak correlation timing between the cortical electrophysiological arousal metric and thalamus hemodynamic response is shifted towards the typical hemodynamic delay. As awakening time prolongs, positive correlation is shifted to an earlier lag and eventually restored to ~ 2 s at ‘A3’, representing reduced cortical sleep-promoting activities as sleep inertia dissipates, while thalamus continues to regulate arousal signaling to promote wakefulness.

This study is the first exploration into the temporal relationship between EEG spectral slope and BOLD response. Recent studies have demonstrated that EEG spectral slope, a scale-free property, can serve as a measure of behavioral output^70–72^, arousal^8^ and selective attention^9^. Therefore, we explored whether EEG spectral slope may reflect cognitive changes as awakening time prolonged. While post-hoc tests did not pinpoint when spectral slope changed between particular imaging sessions, we did find a significant effect by imaging session, suggesting potential fluctuations in cortical neurons’ population firing across sleep inertia stages. Meanwhile, the ACC exhibits strong functional connectivity with frontal, posterior-parietal, and temporal regions^73,74^, and is involved in error detection, attention, goal-directed behavior, and consciousness^75–77^. In light of the fact that sleep inertia manifests reduced cognitive performance and awareness, the potential functional overlap between the ACC and spectral slope raises the possibility of altered coupling during sleep inertia. Indeed, the shift towards an earlier lag between ACC and spectral slope from ‘Pre’ to ‘A2’ may indicate decoupling between the ACC and cortical arousal uniquely during the middle stages of sleep inertia, which ultimately returns to its baseline state by ‘A3’. This is supported by the ~ 6 s peak correlation timing, resembling typical hemodynamic delay.

It was noticed that the prior sleep stage right before awakening may lead to different brain patterns during sleep inertia^14^. Accordingly, we conducted additional NVC analyses by separating the participants into two groups: the N1/N2 group awakened in light sleep stages (n = 15) and the N3/REM group awakened in sleep stages of N3 and REM sleep (n = 6). In the N1/N2 group, we observed similar trends of EEG arousal features (Figure S5) as the main results of Fig. 2, except the alpha-vigilance and spectral slope across presented significant difference across inertia conditions (Friedman test *p* = 0.03 and 0.01, respectively). Similarly, the peak-correlation time lag between thalamus and alpha-vigilance in the N1/N2 group (Figure S6A) resembled Fig. 3B with additional statistical significance in the post hoc comparison between Pre and A1 (*p* = 0.04, FDR-corrected). The ACC-spectral slopes in the N1/N2 group (Figure S6B) remained the same statistical findings in Fig. 3D. In the N3/REM group, we did not observe significant changes due to the small sample size. Collectively from the between-group observations, we noticed that the dynamic changes of EEG-fMRI coupling during sleep inertia was more consistent among the participants awakened from the light sleep, whereas the N3/REM group showed high variability in the EEG-fMRI coupling. Future studies are warranted to confirm the impact of prior sleep stages before awakening on the sleep inertia effects in terms of the coupling between EEG and BOLD signals.

### Limitations and future directions

Since the main objective is to probe the transient signals in sleep inertia using EEG-fMRI, we could not collect sufficient subjective reports of wakefulness simultaneously with our neuroimaging sequence. Therefore, we did not have direct behavioral evidence to support the initiation, maintenance, and dissipation of sleep inertia in our experiment. Second, the low correlation values between thalamus and alpha-vigilance in ‘A3’ should also be noted (Fig. 3A). This could have originated from the instruction of eye-open in the resting-state scan, which is known for less fluctuations of arousal during resting wakefulness^57^. The drop in correlation values may be an indication to the dissipation of sleep inertia, but future studies should adopt the eyes-closed approach to better elicit transient arousal fluctuations during resting-state to elucidate our findings here.

## Conclusion

This is the first attempt to quantify the NVC dynamics under three consecutive post-awakening sessions using simultaneous EEG-fMRI. We found that throughout the course of sleep inertia, the EEG features of arousal (theta/beta ratio, spectral slope, and vigilance index) and cognitive ability undergo minor alterations, which potentially explain the transition from maintenance to dissipation of sleep inertia. More importantly, we found that alpha-vigilance and spectral slope would dynamically couple to BOLD-fMRI response in thalamus and ACC depending on the awakening time. Taken together, our results of time-varying EEG-fMRI correlation suggest the dynamic alterations to neurovascular coupling as a potential candidate that dictates neural dynamics during sleep inertia, and paves the path for future investigations into the neurophysiological mechanisms of sleep–wake transitions.

### Supplementary Information

Supplementary Information.

## Data Availability

The data supporting the conclusions of this article will be made available by the request to the corresponding author, Changwei Wu, without undue reservation.
