# Supplementary material for: Traces of EEG-fMRI coupling reveals neurovascular dynamics on sleep inertia
Source: Sci Rep. 2024 Jan 17;14:1537. doi: 10.1038/s41598-024-51694-4 (PMC10794702; doi:10.1038/s41598-024-51694-4)
Supplement: Supplementary file 1 — Supplementary Information. [file 41598_2024_51694_MOESM1_ESM.docx]

**Supplementary Materials**

**Table S1. Sleep statistics for all subjects.**

| **Subject ID** | **TIB** | **TST** | **%N1** | **%N2** | **%N3** | **%REM** | **Awakened Stage** | |
| --- | --- | --- | --- | --- | --- | --- | --- | --- |
| **SI207** | 94.5 | 64.5 | 11.63 | 64.34 | 10.08 | 13.95 | N2 |  |
| **SI208** | 175.5 | 125 | 16.4 | 76.8 | 0.8 | 6 | R |  |
| **SI209** | 78 | 62 | 1.61 | 60.48 | 37.9 | 0 | N3 |  |
| **SI210** | 176.5 | 136.5 | 4.76 | 60.81 | 17.95 | 16.48 | N2 |  |
| **SI212** | 76.5 | 14.5 | 3.57 | 96.43 | 0 | 0 | N2 |  |
| **SI214** | 167.5 | 143.5 | 4.18 | 59.93 | 24.74 | 11.15 | N2 |  |
| **SI215** | 105 | 42.5 | 35.29 | 35.29 | 22.35 | 7.06 | N1 |  |
| **SI216** | 138 | 104.5 | 9.57 | 76.56 | 6.7 | 7.18 | R |  |
| **SI217** | 131.5 | 116 | 7.76 | 43.97 | 40.52 | 7.76 | N2 |  |
| **SI218** | 104.5 | 73.5 | 1.36 | 65.31 | 31.97 | 1.36 | N3 |  |
| **SI220** | 87.5 | 4.5 | 11.11 | 88.89 | 0 | 0 | N1 |  |
| **SI221** | 41.5 | 25 | 8 | 82 | 8 | 2 | N3 |  |
| **SI222** | 49 | 9.5 | 0 | 21.05 | 5.26 | 73.68 | R |  |
| **SI223** | 107.5 | 22.5 | 11.11 | 33.33 | 6.67 | 48.89 | N1 |  |
| **SI226** | 127.5 | 94 | 10.11 | 88.83 | 1.06 | 0 | N2 |  |
| **SI227** | 177 | 149 | 2.68 | 54.7 | 27.18 | 15.44 | N2 |  |
| **SI228** | 121 | 42.5 | 2.35 | 92.94 | 0 | 4.71 | N2 |  |
| **SI229** | 148 | 97.5 | 3.59 | 86.15 | 10.26 | 0 | N2 |  |
| **SI230** | 107.5 | 92 | 15.22 | 43.48 | 41.3 | 0 | N1 |  |
| **SI232** | 77.5 | 69.5 | 26.62 | 29.5 | 38.13 | 5.76 | N1 |  |
| **SI234** | 74.5 | 34 | 7.35 | 42.65 | 23.53 | 26.47 | N1 |  |
| **Mean:** | 112.67 | 72.5 |  |  |  |  |  |  |
| **S.E.M.:** | 8.68 | 9.81 |  |  |  |  |  |  |

**TIB**=Time in Bed (min); **TST**=Total Sleep Time (min); **%N1, %N2, %N3, %REM=**Percentage of time spent in each sleep stage; **Awaken Stage**=Last sleep stage before awakening.


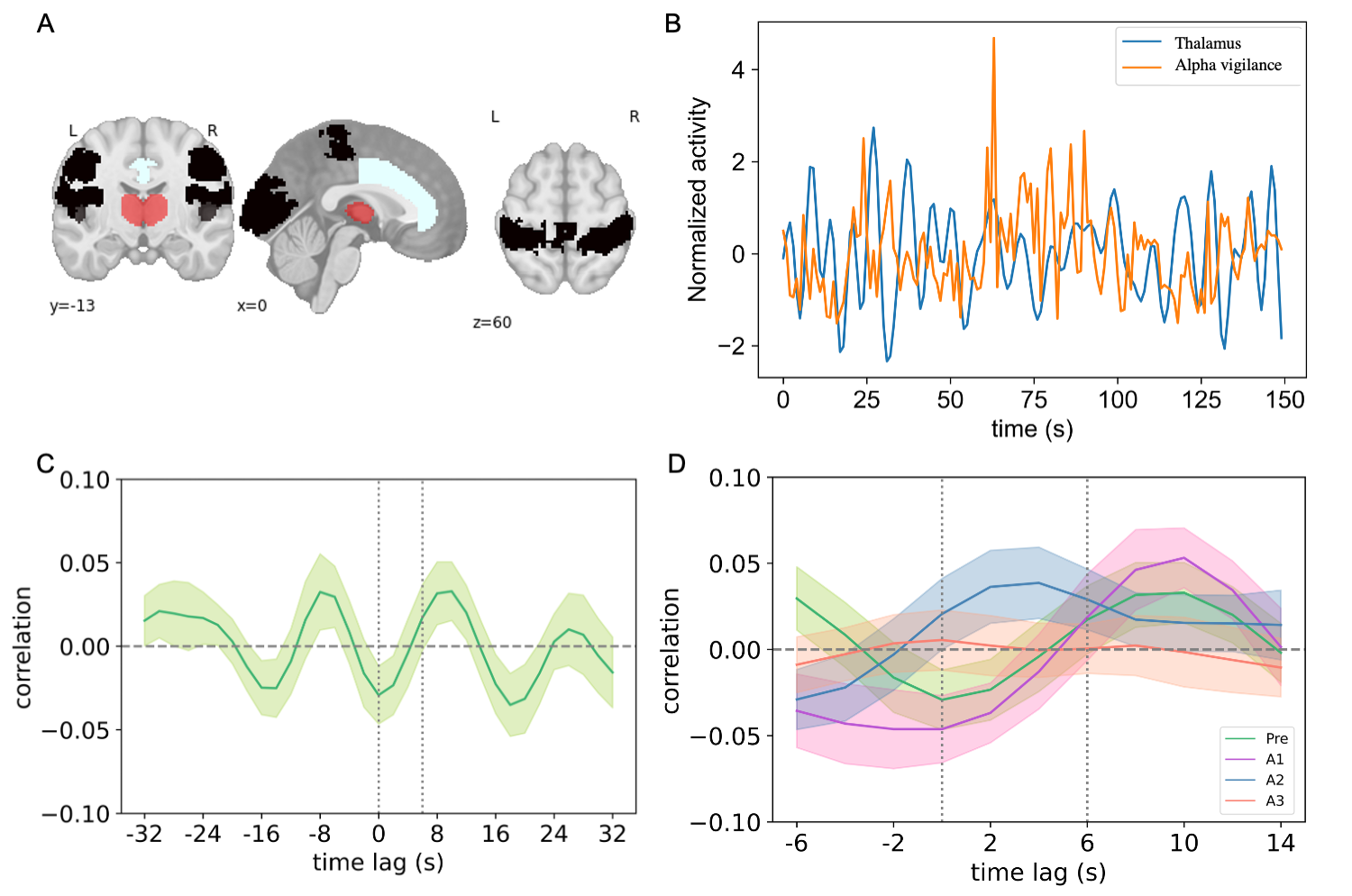


**Figure S1**. **EEG-BOLD cross-correlation patterns. (A)** ROI masks used to extract BOLD time series from ROIs. Dark mask = SM, red = thalamus, cyan = ACC. **(B)** Example time series of thalamus BOLD activity and alpha-vigilance in ‘Pre’ condition from subject 01. Both EEG and BOLD time series were normalized by z-transform for visualization. **(C)** Cyclic cross-correlation pattern between alpha-vigilance and thalamus in ‘Pre’ condition for all subjects. A positive time lag means alpha-vigilance would lead thalamic BOLD signal, and vice versa for negative time lag. **(D)** Alpha-vigilance and thalamus BOLD signal cross-correlated across all four imaging sessions. The time-lag ranges were specifically chosen after considering average periodic fluctuations of cross-correlation coefficients (0.05 Hz). Shaded area represents 1 S.E.M., and two vertical lines were drawn at 0s and 6s time lags, with the latter referring to canonical hemodynamic delay.

**
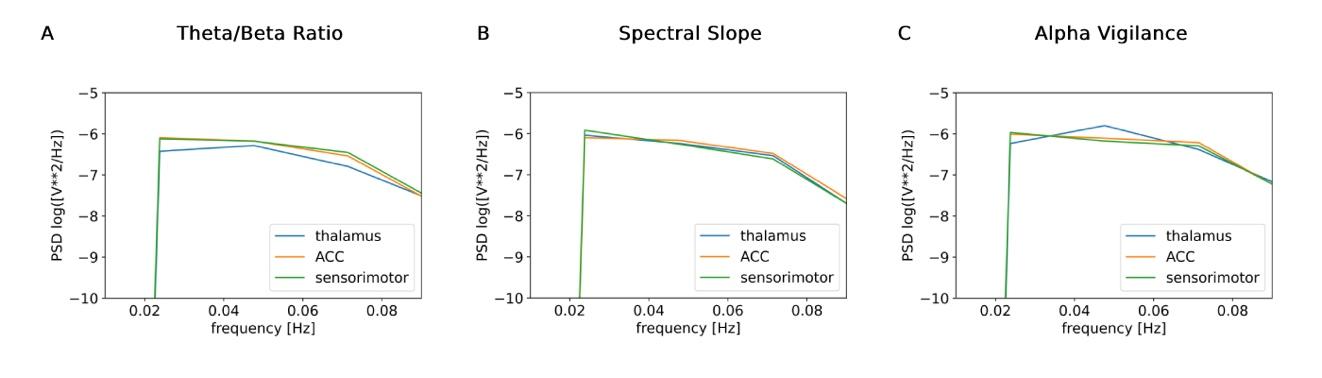
**

**Figure S2. Periodograms of correlation values between EEG metrics and BOLD ROIs. A-C)** Averaged periodograms of correlation values between the 3 prescribed ROIs and theta/beta ratio, spectral slope, and alpha-vigilance, respectively across time lags between -32s to 32s. PSD values underwent logarithmic transformation for better visualization of the ~0.05 Hz peak, which led to considering a 20s window for evaluating time-lag at which peak correlation occurred between EEG features and BOLD ROI time courses.


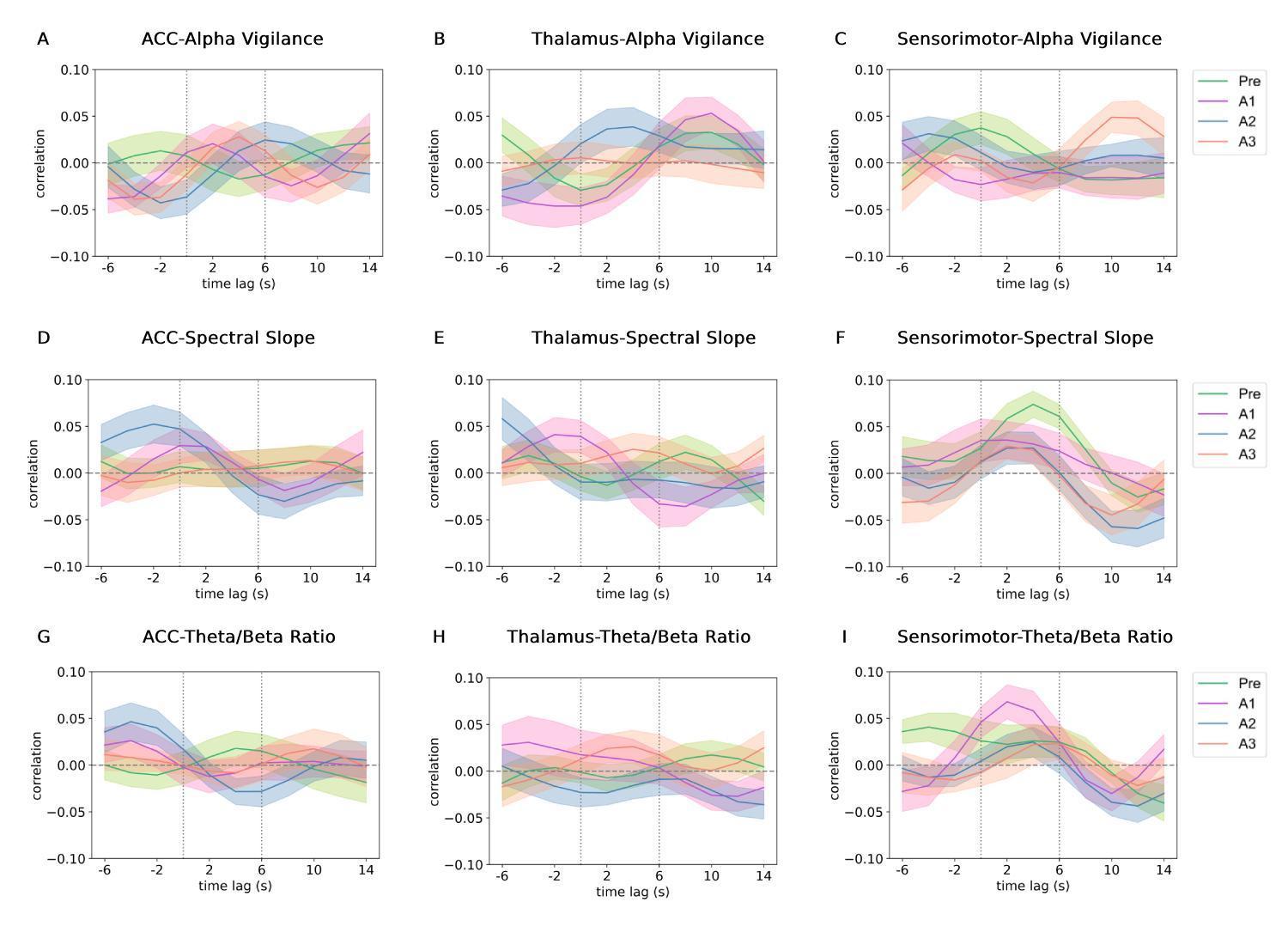


**Figure S3. Cross correlation between EEG metrics and ROIs’ BOLD activity. A-C)** Alpha-vigilance cross-correlated with ROI BOLD activity. **D-F)** EEG spectral slope cross-correlated with ROI BOLD activity. **G-I)** Theta/beta ratio cross correlated with ROI BOLD activity.


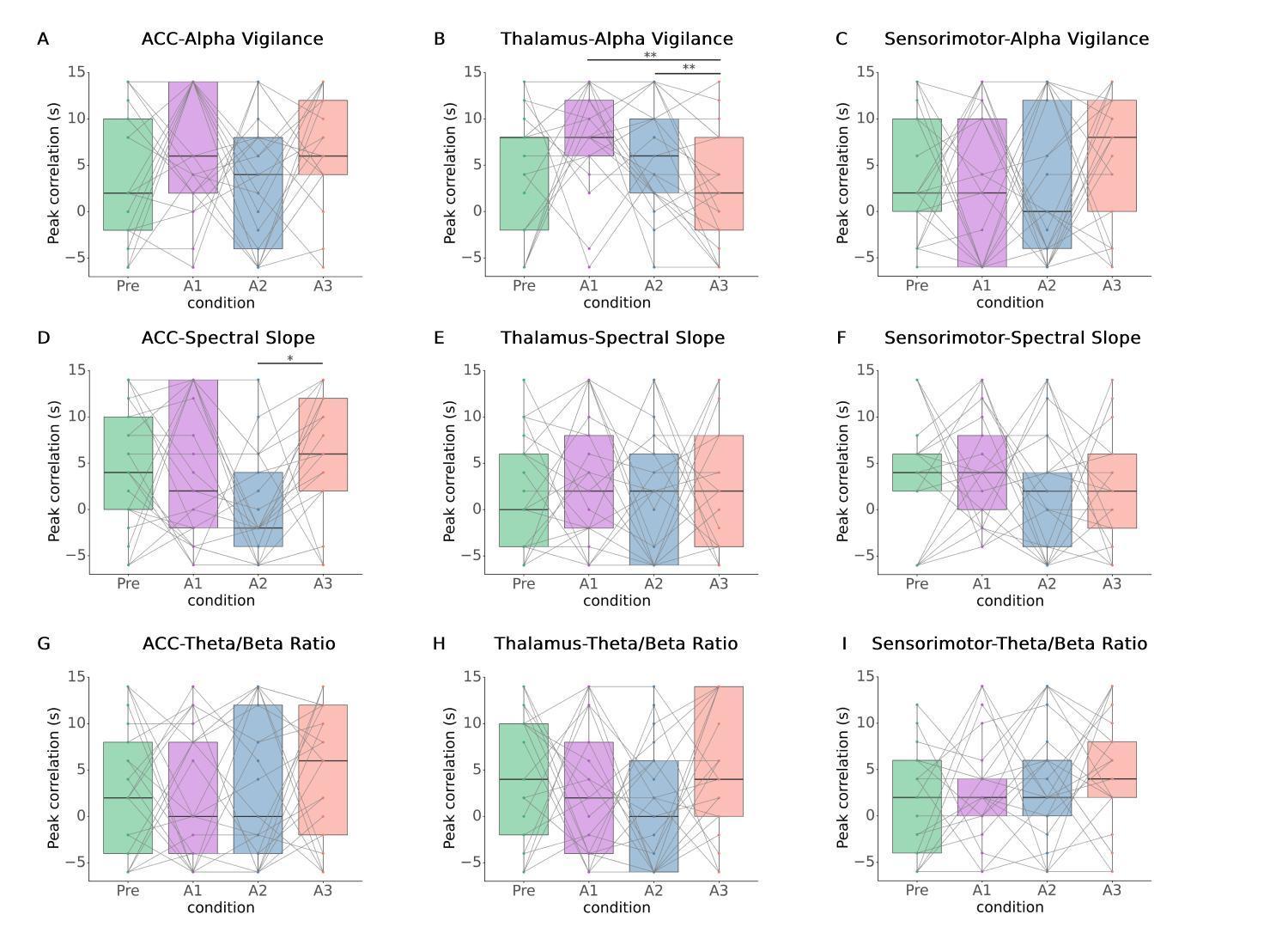


**Figure S4. Time lags of peak positive correlation (range: -6 and 14 seconds) between EEG metrics and ROIs’ BOLD activity across sessions. A-C)** Time lags where maximum positive correlation between alpha-vigilance and ROIs’ BOLD occurred. There is a significant effect by imaging session in thalamus and alpha-vigilance correlation (*p* = 0.02), and A1 and A2’s peak correlation timings are significantly later than A3’s (***p* = 0.01, FDR-corrected). **D-F)** Time lags where maximum positive correlation between EEG spectral slope and ROIs’ BOLD occurred. There is a significant effect by imaging session (*p* < 0.01), where A2 is found to be significantly earlier than A3 (**p* = 0.03, FDR-corrected). **G-I)** Time lags where maximum positive correlation between EEG theta/beta ratio and ROIs’ BOLD occurred. No effect by imaging session was found among all correlations (*p* > 0.1).


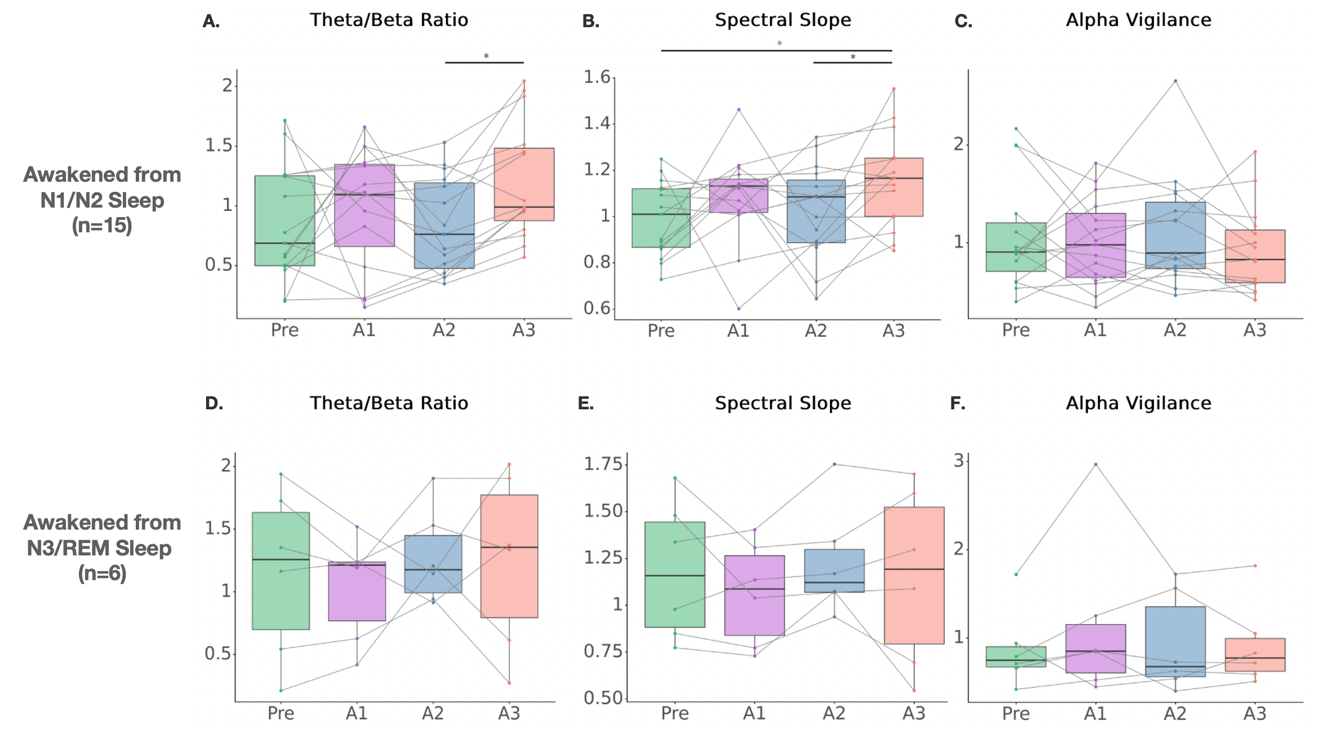


**Figure S5**. **Demonstration of EEG arousal features across four imaging sessions, separated into two groups according to the prior sleep stage before awakening. (A)** For the participants awakened from the light sleep stages (n=15), averaged theta/beta ratio showed significant changes across the four sessions (Friedman test *p* = 0.03), where theta/beta ratio in A3 is higher than A2 in *post hoc* test (Wilcoxon sign-rank test, **p* = 0.01, FDR-corrected). **(B)** For the participants awakened from the light sleep stages, spectral slope was found to fluctuate significantly across imaging sessions (Friedman test *p* = 0.01), where A3 is significantly higher than A2 and Pre in *post hoc* test (Wilcoxon sign-rank tests **p* = 0.04, FDR-corrected). **(C)** No significance was found for alpha-vigilance across imaging sessions (Friedman test *p* > 0.1). **(D-F)** For the participants awakened from the sleep stages of N3 and REM (n=6), there is no significant difference was found for all three arousal features across the four sessions (Friedman test *p* > 0.1).


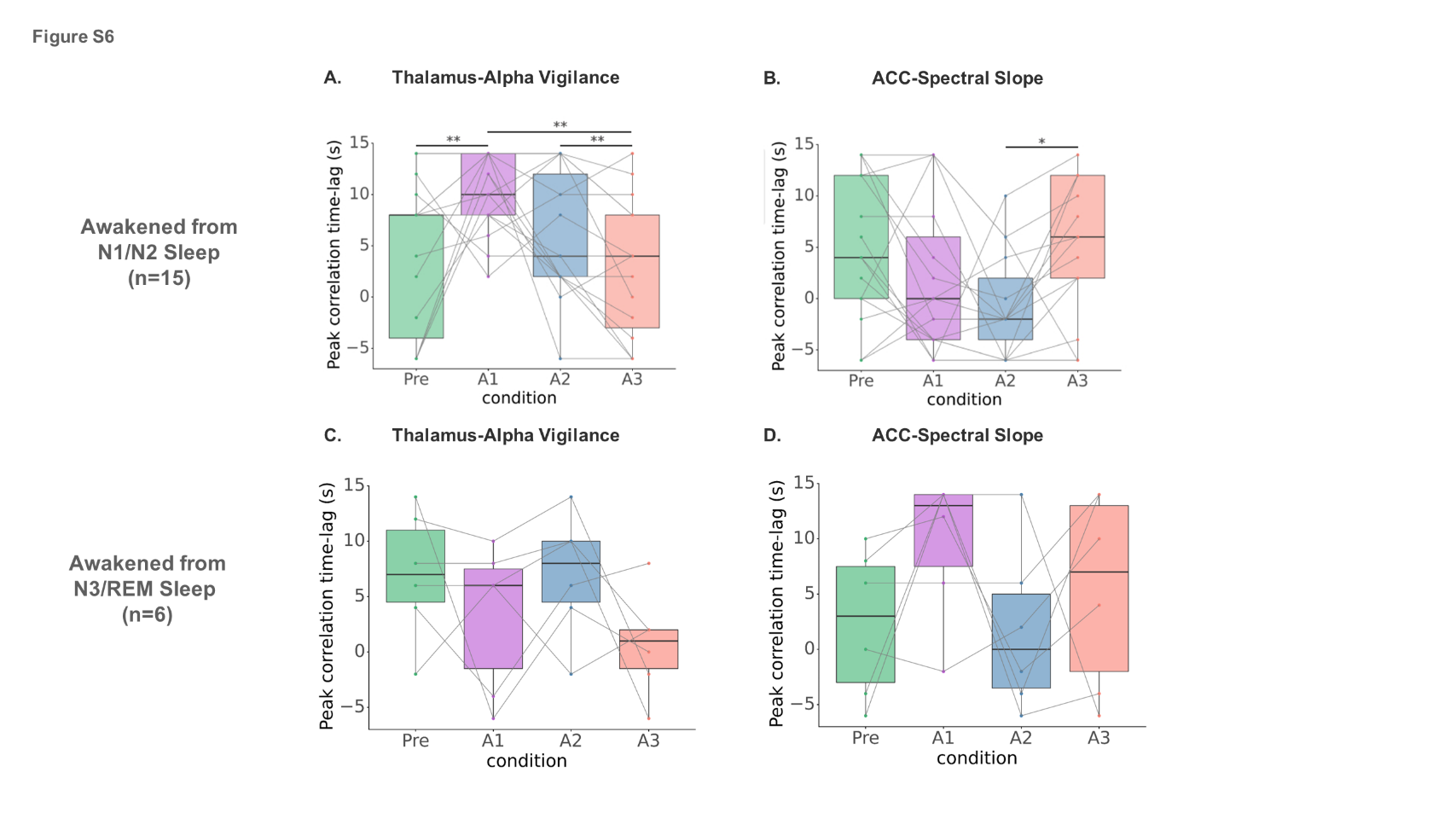


**Figure S6. Demonstration of peak-correlation time lags between EEG metrics and BOLD activity across the four sessions, separated into two groups according to the prior sleep stage before awakening. (A)** For the participants awakened from the light sleep stages (n=15), the peak correlation time lags between thalamus and alpha-vigilance shows significant difference across sessions (*p* = 0.02). The peak-correlation timings of A1 and A2 are significantly later than those of A3, and the time lag of A1 is also significantly later than that of Pre (***p* = 0.04, FDR-corrected). **(B)** For the participants awakened from the light sleep stages, the peak correlation time lags between ACC and EEG spectral slope shows significant difference across sessions (*p* = 0.02), where A2 is found to be significantly earlier than A3 (**p* = 0.04, FDR-corrected). **(C-D)** For the participants awakened from the sleep stages of N3 and REM (n=6), the peak-correlation time lags between thalamus and EEG alpha-vigilance and the peak-correlation time lags between ACC and EEG spectral slope did not show significance across sessions (*p* > 0.1).
